# Direct non-cell autonomous Pax6 activity regulates eye development in the zebrafish

**DOI:** 10.1186/1749-8104-2-2

**Published:** 2007-01-17

**Authors:** Brigitte Lesaffre, Alain Joliot, Alain Prochiantz, Michel Volovitch

**Affiliations:** 1 Development and Neuropharmacology, CNRS UMR 8542 and Ecole Normale Supérieure, rue d'Ulm, 75230 Paris Cedex 05, France; 2 Homeoprotein cell biology, CNRS UMR 8542 and Ecole Normale Supérieure, rue d'Ulm, 75230 Paris Cedex 05, France

## Abstract

**Background:**

Modifications in *Pax6 *homeogene expression produce strong eye phenotypes. This suggested to us that eye development might be an appropriate model to verify if homeoprotein intercellular passage has important functions in early development. Similar to other homeoproteins, Pax6 has two domains that enable secretion and internalization by live cells and, thus, intercellular passage. In principle, a straightforward way to test the hypothesis would be to mutate one of the two sequences to produce a 'cell autonomous only' Pax6. However, this was not possible because these sequences are in the homeodomain and their modification would affect Pax6 transcriptional properties. We have thus developed an approach aimed at blocking Pax6 only in the extracellular milieu of developing zebrafish embryos.

**Results:**

A first strategy was to inject a one-cell embryo with a mRNA encoding a secreted single-chain anti-Pax6 antibody. A second, complementary, strategy was to inject a Pax6 antibody in the blastula extracellular milieu. In both cases, 'dissymmetric eyes', 'one eye only' and 'no eye' phenotypes were produced. In most cases, lens phenotypes paralleled retina malformations. Although eye phenotypes were analyzed 30 hours post-fertilization, there was a strong correlation between early eye field asymmetry, early asymmetry in *Pax6 *expression and later-occurring eye malformations. Several controls were introduced, demonstrating that the effect is specific to Pax6 and cannot be explained by intracellular antibody activities.

**Conclusion:**

This study supports the hypothesis that the Pax6 transcription factor is also a signaling molecule with direct non-cell autonomous activity.

## Background

Eye formation is one of the most popular models used to study the development and evolution of sensory systems [1]. Similarities exist between vision apparatus across species, leading to the two hypotheses of convergent evolution versus monophyletic origin [2]. This system is also widely used for induction studies due to the inductive interactions that take place between neural and non-neural tissues in the course of eye development. Spemann was the first to propose that the interaction between the neural fold and the surface ectoderm is at the origin of the induction of lens formation [3-5]. This induction between the two tissues has been challenged and it has been hinted that all species may not use identical strategies to develop a visual apparatus [6-8]. Although these evolutionary and developmental issues are not fully resolved, it is now largely accepted that certain shared genetic pathways play important functions in the development of vision across species.

The main breakthrough in the understanding of how the eye has evolved and develops has come from genetic analysis, in particular the identification of Pax6 as a key transcription factor for eye formation [9]. Indeed, *Pax6 *loss of function, partial or total, leads to abnormal eye development in all species, including human [2,10-12]. Conversely, excessively high expression leads to eye malformation [13-16] or to the spectacular formation of ectopic eyes [17,18]. The expression of *Pax6 *in the 'vision apparatus' of all species, as well as the possibility to induce eye formation in the fly by expressing vertebrate *Pax6*, also strongly supports the idea that, in spite of multiple variations on the theme, there exists a common genetic pathway in which *Pax6 *or *Pax6*-like genes play a major role [19,20].

Interestingly, *Pax6 *is expressed in both neural and surface epithelia that will give birth to retina proper and retina pigmented epithelium (RPE) for the former and to lens and cornea for the latter. This raises two main questions regarding, firstly, the extent of the retina territory within the neural fold and, secondly, the mechanisms of induction between the neural tissue and the surface epithelium (lens induction). In both cases, it has been established that the genetic pathways involve several transcription factors and growth factors of the Fibroblast Growth Factor (FGF) and Bone Morphogenetic Protein (BMP) families [5,21-26].

In the present study, we wanted to investigate the possibility that Pax6 could act as both a cell autonomous transcription factor [5] and a non-cell autonomous signaling factor [27,28] capable of inducing *Pax6 *expression after intercellular passage [29-32]. The possibility that a transcription factor could have both cell autonomous and non-cell autonomous activity originates from the presence within the homeodomain of a majority of homeoprotein transcription factors, including *Pax6*, of two short sequences that enable secretion and internalization and, thus, intercellular passage [33-35]. Based on the phenotypes obtained following the *in vivo *expression of Pax6 antibodies in the intercellular space, we propose that, in addition to other regular inducers and growth factors, Pax6 acts as a signaling molecule during eye development.

## Results

**Figure 1:**
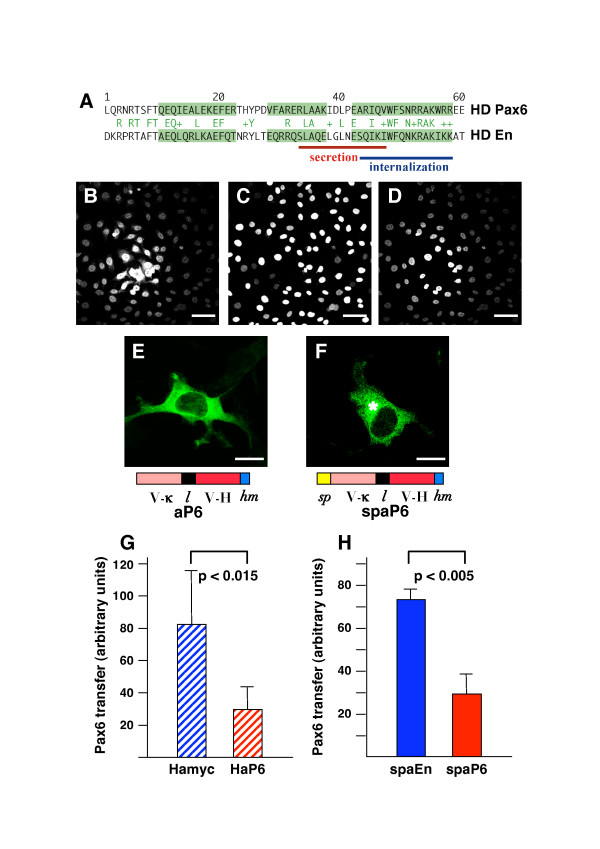
Pax6 intercellular transfer is blocked by extracellular anti-Pax6 antibodies. **(a) **Sequence alignment of Pax6 and En2 homeodomains. Consensus amino acids are indicated in green letters between the two sequences; alpha helices are shaded. Amino acids involved in secretion and internalization are underlined (in red and blue, respectively). **(b-d) **COS-7 cells were transfected with a *Pax6 *expressing plasmid and co-cultured with HeLa cells marked by a GFP-H2b fusion. Pax6 transfer was monitored in every optical field by quantifying the Pax6 signal (overall signal shown in (b)) only in GFP-expressing HeLa cells (shown in (c)), giving (in (d)) the transfer signal as described in Materials and methods. Scale bar = 60 μm. **(e,f) **Subcellular localization of single-chains aP6 (e) or spaP6 (f) transiently expressed in COS-7 cells. Schematic representations of the antibodies are presented: V-k, kappa chain variable region; l, linker; V-H, heavy chain variable region; hm, his-myc tag. Whereas aP6 is detected throughout the cytoplasm and in the nucleus, spaP6 is restricted to vesicular structures and the Golgi apparatus (asterix). Scale bar = 10 μm. **(g,h) **Inhibition of Pax6 intercellular transfer. **(g) **COS-7 cells expressing *Pax6 *were cultured together with HeLa cells in medium supplemented with anti-myc hybridoma (Hamyc) or anti-Pax6 hybridoma (HaP6). **(h) **COS-7 cells co-expressing *Pax6 *and secreted single-chain antibodies against either Engrailed (spaEn) or Pax6 (spaP6) were cultured together with HeLa cells. In (g,h), Pax6 transfer to HeLa cells (analyzed as described in Materials and methods, in three independent experiments) was significantly reduced by hybridoma addition (p < 0.015) or single-chain co-expression (p < 0.005).

The presence within the Pax6 sequence of the two small domains allowing homeoprotein internalization and secretion (Figure 1a) [33] led us to investigate Pax6 intercellular passage. As shown in Figure 1b–d, intercellular transfer from *Pax6*-transfected COS cells towards HeLa cells was verified by quantifying Pax6 detection within recipient HeLa cells.

In principle, the most elegant way to identify homeoprotein non-cell autonomous activities *in vivo *would be to replace the endogenous gene by a version mutated for intercellular transfer. This is, unfortunately, impossible because the two transport sequences that should be mutated are within the DNA-binding domain (homeodomain), and thus also instrumental for cell autonomous activities. This is why we developed a strategy based on the possibility of using specific anti-Pax6 antibodies to trap this transcription factor as it travels between cells.

We used the immunoglobulin light and heavy chains expressed by the anti-Pax6 hybridoma to clone a cDNA encoding an anti-Pax6 single chain antibody. Two versions were made, differing by the absence (aP6) or presence (spaP6) of a secretion signal peptide. As shown in Figure 1f, spaP6 gains access to the Golgi and is present within vesicles, thus showing a distribution strikingly different from that of its non-secreted counterpart, aP6, which is detected throughout the cytoplasm (Figure 1e). Figure 1e,f also illustrates that, in contrast to aP6, spaP6 is absent from the nucleus, suggesting that very few antibody molecules, if any, escape from the secretion pathway. To verify the transfer blocking activity of the antibodies, we first added the monoclonal anti-Pax6 antibody, or a monoclonal anti-myc antibody as a control, into the medium of co-cultured *Pax6*-expressing COS-7 cells and marked HeLa cells (see Materials and methods). Figure 1g illustrates that anti-Pax6 antibodies in the culture medium significantly reduce intercellular transfer between expressing and non-expressing cells, as compared to control. We then co-cultured HeLa marked cells with COS-7 cells co-expressing Pax6 and secreted single-chain anti-Pax6 (spaP6) or anti-Engrailed (spaEn) antibodies. Figure 1h illustrates that spaP6, and not spaEn, blocks Pax6 transfer.

**Figure 2:**
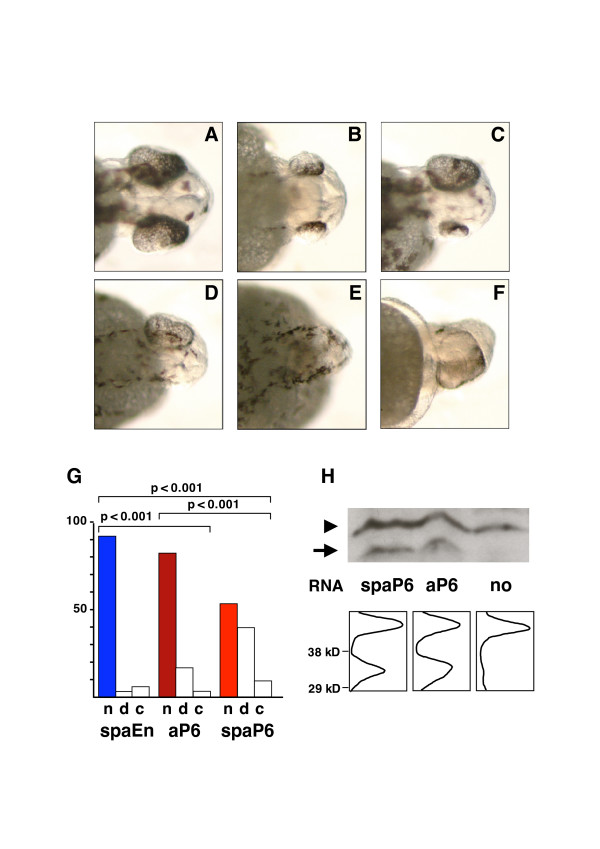
Eye phenotypes induced by secreted anti-Pax6 single-chain antibody. **(a-f) **Eye phenotypes observed at 30 hpf after injection of secreted single-chain anti-Pax6 (spaP6) mRNA into one cell embryos. **(a) **Normal, **(b) **small eyes, **(c) **dissymmetric eyes, **(d) **single eye, **(e) **no eye, **(f) **cyclops. **(g) **Quantification (percentage) of phenotypes: n, normal; d, dissymmetric, reduced and absent eyes (pooled); c, cyclops. Comparison between secreted anti-Pax6 (spaP6, n = 221, red), secreted anti-En (spaEn, n = 251, blue) and non-secreted anti-Pax6 (aP6, n = 178, dark red). Differences between spaP6 and spaEn or aP6, as well as between aP6 and spaEn, are statistically significant (p < 0.001, χ^2 ^tests). **(h) **Quantification of single-chain mRNA expression. Top: western blot of protein extracts from embryos injected with the indicated RNAs (no : no RNA); the arrow indicates a single-chain antibody signal at approximately 34 kDa, and the arrowhead the cross-hybridizing protein species used for calibration. Bottom: densitometric scans of western signals indicate aP6 expression (ratio 1:1 with the endogenous signal) is at least as high as spaP6 expression (ratio 0.7:1 with the endogenous signal).

The monoclonal anti-Pax6 antibody had been produced against the amino-terminal Pax6 domain, which is highly conserved in all Pax6 sequences. Having verified that this antibody recognizes zebrafish Pax6 proteins (not shown), capped mRNAs encoding single chain antibodies were injected into the zebrafish embryo at the one cell stage. Eye morphologies observed 30 hours post-fertilization (hpf) and induced by the secreted anti-Pax6 single-chain antibody (spaP6) are illustrated in Figure 2a–f. The main phenotype observed is a decrease in eye size, either symmetrical (as in Figure 2b) or asymmetrical (Figure 2c). In extreme cases we noticed the absence of one eye (Figure 2d), or even of both eyes (Figure 2e). Cyclops were also observed (Figure 2f), but the percentage of animals with this phenotype was similar, independent of the nature of the mRNA injected in the egg (Figure 2g). In control experiments (spaEn), 91% of the embryos had symmetrical eyes, 3% showed dissymmetry and 6% were cyclops (Figure 2g, left). In strong contrast, the *in vivo *expression of spaP6 resulted in a ten-fold increase (40% of the embryos) in the number of dissymmetric eyes or small eyes, with no significant changes in the number of cyclops (Figure 2g, right).

Because the latter eye phenotypes could result from the cell autonomous activity of spaP6 antibodies escaping from the secretion pathway, we compared the effects of injecting spaP6 and aP6 capped mRNAs. Figure 2g (middle) shows that antibodies deprived of signal peptide also have an effect on eye development, but that this effect (15% of the embryos show eye dissymmetry) is significantly smaller than that of secreted anti-Pax6. The significant difference between aP6- and spaP6-induced phenotypes did not result from differences in expression as illustrated in Figure 2h, in which amounts of antibodies expressed at gastrulation are compared. Most likely, the difference between aP6- and spaP6-induced phenotypes is due to the known instability of many intracellular antibodies after disulfide bonds have been reduced by the high intracellular glutathione content [36,37]. This series of experiments thus strongly suggests that the eye phenotype observed after spaP6 mRNA injection reflects a decrease in Pax6 direct non-cell autonomous activity.

**Figure 3:**
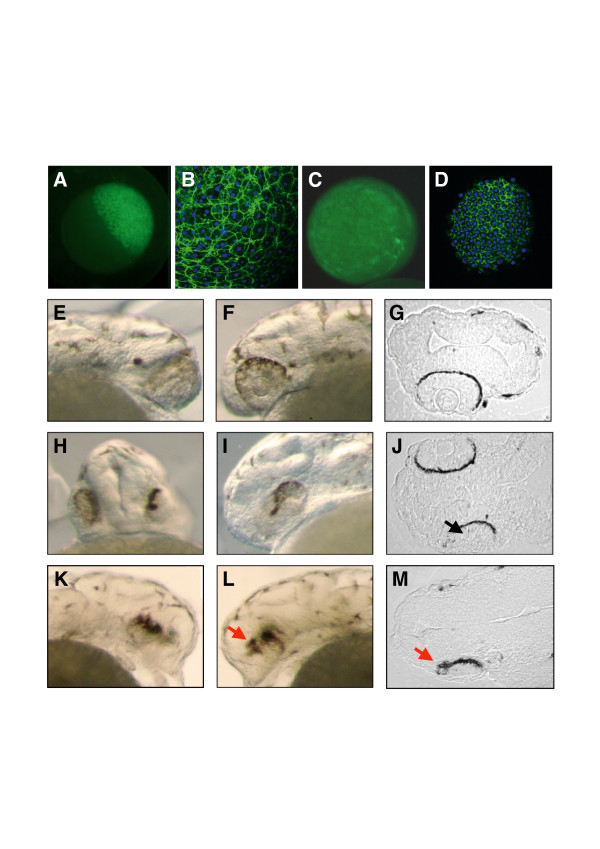
Eye phenotypes induced by intercellular injection of anti-Pax6 monoclonal antibody. **(a-d) **Intercellular distribution of FITC-labeled anti-mouse antibody injected at the blastula stage. Embryos were analyzed *in toto *(a,c) or using confocal microscopy (b,d) either 1 hour after injection (a,b) or at the shield stage (c,d). Note that the staining is concentrated in the intercellular space and is virtually absent from the cell interior. **(e-m) **Prototypical eye phenotypes (quantifications in Figure 4): Embryo 1 (e-g) has a severely reduced right eye (lateral view (e)) compared with left eye (lateral view (f)) and no right retina or lens (section in (g)). Embryo 2 (h-j) has a strongly reduced left eye (frontal view in (h), lateral view in (i)). The horizontal section (j) confirms the phenotypes and indicates that the left lens is also reduced (arrow). Embryo 3 has two reduced eyes with disorganized morphologies (lateral views in (k,l)) and a reduced and misplaced left lens (red arrow in (l,m)). In all horizontal sections, rostral is to the left.

**Figure 4:**
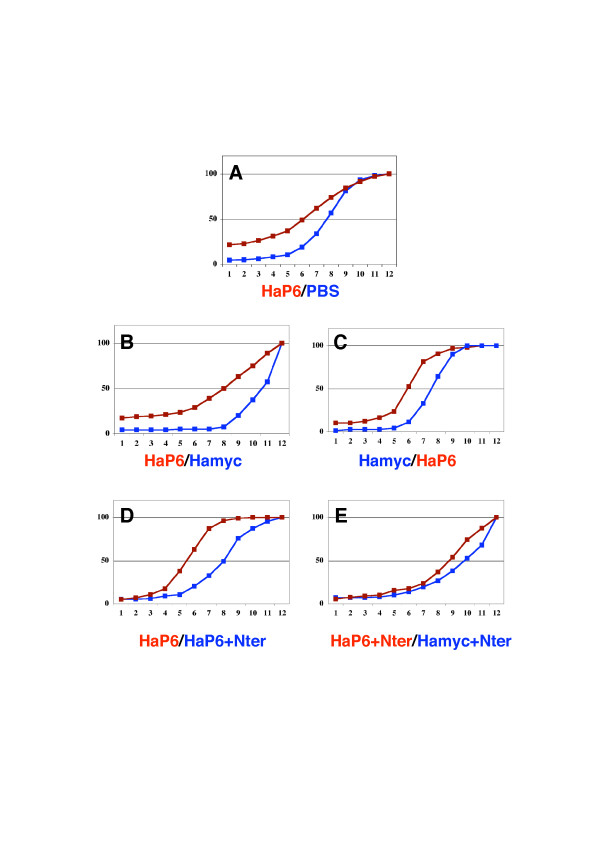
Quantification and specificity of monoclonal anti-Pax6 effects. Eye area (measured in arbitrary units using Lucia G software, and distributed among 12 classes) of embryos injected at the blastula stage and presented as cumulative frequency plots. Reagents were always compared pair-wise on a single clutch. **(a) **Monoclonal anti-Pax6 (HaP6, number of eyes measured n = 161) versus vehicle (phosphate-buffered saline (PBS), n = 164). **(b) **HaP6 injected first in one half of the clutch (n = 128) versus monoclonal anti-myc (Hamyc, n = 120) injected immediately after in the second half of the clutch. **(c) **Hamyc (n = 70) versus HaP6 (n = 98), injected in the reverse order to (b). **(d) **HaP6 (n = 216) versus HaP6 pre-incubated with the amino-terminal arm of Pax6 (HaP6+Nter, n = 132). **(e) **HaP6 pre-incubated with the amino-terminal arm of Pax6 (HaP6+Nter, n = 198) versus Hamyc pre-incubated with the amino-terminal arm of Pax6 (Hamyc+Nter, n = 138). Differences are statistically significant for (a-d) (p < 0.0001, Mann-Whitney tests).

To further verify the latter possibility, we injected the hybridoma supernatant (HaP6) in the intercellular space at the blastula stage. Using a fluorescein isothiocyanate (FITC)-antibody, we first verified that antibodies injected in the blastula diffuse between the cells and are not internalized (Figure 3a–d). The distribution of the antibody, followed one hour after injection (Figure 3a,b) or at the shield stage (Figure 3c,d), was predominantly, if not exclusively, extracellular. We then injected HaP6 versus vehicle, HaP6 versus HaMyc (anti-myc supernatant) or HaP6 versus HaP6 pre-incubated with the epitope recognized by the antibody (amino-terminal domain of Pax6). Figure 3 illustrates the prototypic phenotypes that were obtained (quantitative analysis in Figure 4) for three embryos when HaP6 was injected at the blastula stage. Eyes are either highly reduced or dissymmetric. Horizontal sections (Figure 3g,j,m) confirm eye reduction and dissymmetry but also show that lenses are either absent (Figure 3g) or smaller and/or displaced (Figure 3j,m). Such phenotypes were never observed in any of the controls and are similar to those observed after the injection of capped spaP6 mRNAs into the egg.

The cumulative plots of eye sizes, not considering asymmetry (all eyes are included, two eyes per animal) are presented in Figure 4. Clearly, HaP6 extracellular antibody has a strong effect on eye size (Figure 4a) that cannot be reproduced with the anti-myc antibody (Figure 4b). To ascertain that this differential effect was not due to a difference in timing, we compared clutches in which the first batch of embryos was injected either with the myc or with the Pax6 antibody, and found no difference (compare Figure 4b and 4c). We also verified that pre-incubating the antibody with the amino-terminal Pax6 epitope abolished the effect on eye size distribution only when the embryos were injected with anti-Pax6 antibodies (raised against this epitope) (Figure 4d,e). This series of results confirm those obtained with the single-chain encoding mRNAs and strongly suggest that full eye development requires extracellular Pax6 expression.

**Figure 5:**
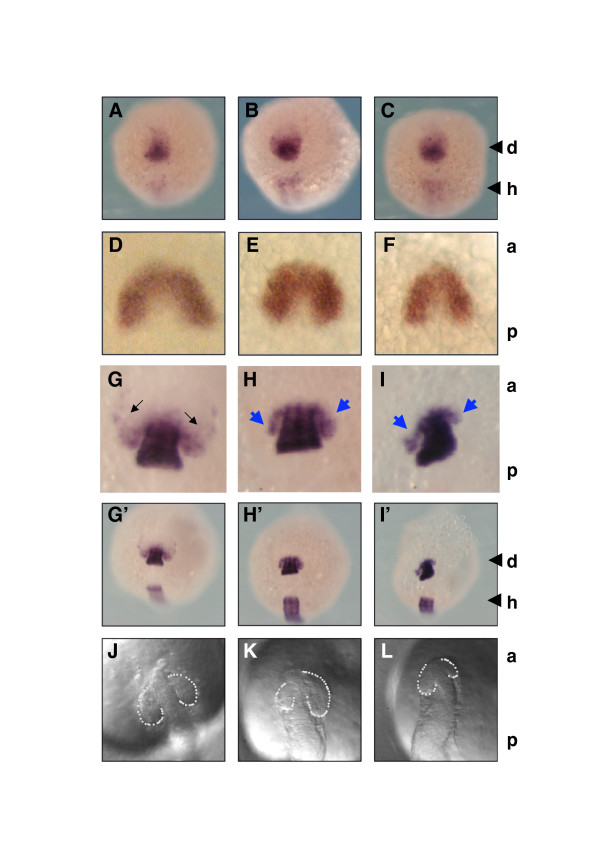
Anti-Pax6 perturbs early *Pax6 *expression in the eye territory. **(a-i) ***Pax6 *(a-c and g-i') or *Rx3 *(d-f) *in situ *hybridization (ISH). Zebrafish embryos were injected at the blastula stage with vehicle (phosphate-buffered saline (PBS) in (a,d,g,g',j) or monoclonal anti-Pax6 (b,c,e,f,h,i,h',i'). (g'-i') Lower magnification versions of (g-i). Arrows indicate symmetric (black) and asymmetric (blue) distributions. ISH was performed at 0 to 3 somites (a-f), or 15 to 18 somites (g-i'). (j-l) Eye fields were photographed on live embryos at about 10 somites, after injection at the blastula stage with PBS (j) or monoclonal anti-Pax6 (k,l). d, diencephalon; h, hindbrain; a, anterior; p, posterior. N = 36 (a); n = 97 (b,c); n = 25 (d); n = 144 (e,f); n = 23 (g); n = 43 (h,i); n = 25 (j); n = 48 (k,l).

Figures 2, 3 and 4 illustrate and quantify the effect, at 30 hpf, of injections done much earlier, either at the one cell stage (capped mRNA; Figure 2) or in the blastula (antibodies; Figures 3 and 4). This does not allow one to verify the stage at which the phenotypes first appear. To address this point we analyzed different landmarks of the eye field between 0 to 3 and 15 to 18 somite stages. At the earliest stage (zero to three somites) no difference in *Pax6 *expression patterns was visible following HaP6 injection in the blastula (Figure 5a–c). The same result was obtained with Rx3, a more extensive marker of very early eye territory (Figure 5d–f). In contrast, antagonizing Pax6 passage led to a clear dissymmetry in *Pax6 *expression in the eye field at the 12 to 15 (not shown) and 15 to 18 somite stages (Figure 5g–i, blue arrows) but not, or not conspicuously, in the other domains where Pax6 is also expressed, for example, the diencephalon and hindbrain (d and h in Figure 5g'–i').

Finally, to verify if the early and late phenotypes were correlated, we observed the eye field of 48 embryos having between 10 and 15 somites, when asymmetry is first visible in the eye field of embryos injected at the blastula stage (Figure 5j–l), and followed the eye development of each individual embryo. All embryos with normal and symmetrical eye fields developed with normal eyes. Among 15 embryos with small but symmetrical eye fields, 3 recovered completely, 3 matured with small eyes, 5 with dissymmetric eyes and 4 lost both eyes completely. Of 15 embryos with dissymmetric eye fields, one developed two small eyes, 2 matured with dissymmetric eyes and 12 developed no eye at all. Finally, out of 14 embryos with no visible eye field, one developed asymmetric eyes and the 13 others never developed any eye. We can thus conclude that the effect of the presence of anti-Pax6 antibodies in the intercellular space takes place early in development, probably before the six somite stage, as soon as the optic vesicle starts to form. In addition, it seems that the type of phenotype seen at an early stage is, in general, confirmed by the abnormality seen at later stages.

## Discussion

In the present study we have verified that, as expected from sequence analysis, Pax6 is capable of intercellular transfer *in vitro *(Figure 1) [34,35]. Our *in vivo *results support the idea of a direct non-cell autonomous activity of Pax6, but say nothing concerning the importance of its capture by abutting cells. Although we favor the hypothesis that the non-autonomous activity reported here requires Pax6 internalization, we do not preclude the possibility of Pax6 also having, or only having, an extracellular activity not requiring internalization.

As already mentioned, because the sequences necessary for homeoprotein transfer are in the homeodomain [33], we could not modify them to generate transgenic animals expressing 'cell autonomous only' proteins. This is why we applied an extracellular antibody strategy. Because of their sensitivity to intracellular glutathion, single chain antibodies are often unstable within the cell and deprived of strong intracellular activities [36,37].

Intracellular instability of single chain anti-Pax6 antibody was indirectly confirmed by the present data, where single chain antibodies devoid of signal peptide, and thus purely intracellular, were far less active than the secreted ones, though the two constructs encoded the same variable domain sequences and were expressed to the same level (Figure 2). This result makes it unlikely that the biological effects of spaP6 (the secreted form) can be explained by the escape of the antibody from the secretion pathway. The latter point was further confirmed by the absence of spaP6 in the nucleus of transfected cells (as opposed to aP6) and by experiments in which the full antibody (from the hybridoma used to clone aP6 and spaP6) was injected in the blastula (Figure 3). The similarities in phenotypes obtained with either strategy (expression or injection) reinforce the interpretation that interfering with direct non-cell autonomous Pax6 activity induces abnormal eye development.

The eye phenotype was primarily a reduction in eye size, from partial to total. This reduction was sometimes symmetrical but one eye was often more affected than the other. A likely explanation is that expressed or injected antibodies are not evenly distributed. Indeed, following injection, the mRNAs could accumulate preferentially in one blastomere or in only one of the first two blastomeres if injected just before the first division. It is not unlikely, in the case of antibody injection in the blastula, that the concentration of antibodies is not homogeneous or does not remain so. In fact, an uneven distribution is visible in Figure 3d, which was taken at the shield stage (confocal section made at 50% epiboly).

Also striking is the fact that a defect in the retina is often accompanied by an absence of lens or the development of a small and/or ectopic lens. The lens and retina phenotypes could be independent since *Pax6 *is expressed in both structures. Alternatively, a defect in the neural retina may have consequences for lens induction or development. In the context of a role of the retina in lens induction, we cannot preclude that Pax6 secretion between the neural fold and the surface epithelium participates in lens induction. This indeed does not eliminate the possibility that classic inducers are under Pax6 cell autonomous transcriptional control [5,21-26,38,39].

The first sign of antibody activity is the abnormal pattern of *Pax6 *expression in the eye field (Figure 5h,i). Compared to controls, the domain of *Pax6 *expression is smaller on one or both sides. Several explanations seem plausible that are not mutually exclusive. One is that Pax6 is a non-cell autonomous regulator of cell death/survival [40] and that blocking its passage reduces eye size through local cell death. A second is that Pax6 is a non-cell autonomous regulator of cell division and that blocking its passage blocks the number of progenitors in the eye field or forces their premature differentiation [16,41,42]. A third is that secreted Pax6 activates its own transcription, either indirectly or after internalization and transport to the nucleus, thus inducing the extension of the eye field [31]. The third possibility, which may provide a molecular mechanism for the concept of homeogenetic induction [43,44] is indeed compatible with the two others since the induced Pax6 could then act as a cell-autonomous regulator of cell survival, division or commitment. Although we have, at this stage, no reason to exclude any scenario, non-autonomous self-activation at the transcription level is compatible with the fact that Pax6 activates its own transcription [29-32].

Interestingly, many studies suggest that, early in development, several homeoproteins act in pairs to position borders within the neuroepithelium. Among several examples, it was shown that a quantitative disequilibrium between Emx2 and Pax6 results in a change in the size of primary sensory areas with no modification in the total surface of the neuroepithelium [45-47]. The same observation was made for the position of the isthmus, which depends on the respective levels of expression of Otx2 and Gbx2 [48,49]. Given that, within a pair, each of these proteins activates its own transcription and down-regulates that of the other, the non-cell autonomous activity described here might provide a parsimonious and simple mechanism for establishing borders.

At the low level of precision permitted by our biological model, it seems that the antibodies only affected eye development and not other structures where the transcription factor is also expressed (Figure 5g–i). Similarly, the anti-Engrailed single chain antibody, taken as a control, had no visible influence on brain morphology (Figure 2g), even though Engrailed-1 and Engrailed-2 are two secreted homeoproteins [35]. A possible explanation is that the changes are too small to be really identified in our experimental model, possibly because they happen later, when the antibodies have already been degraded and diluted. Another possibility is that redundant signaling mechanisms have been selected that are capable of buffering the phenotype. If so, it could be that *Pax6 *and the eye are particularly suitable to investigate this novel signaling mechanism, not only because eyes are highly variable structures but also because *Pax6 *is a major player in eye induction and development, as demonstrated by the development of eyes upon *Pax6 *expression in ectopic localization, both in the fly and in the vertebrates [17,18].

To pursue this line of thought in an evolutionary context, homeoprotein transfer may have been an early mode of signal transduction used for the exchange of positional information in the first multi-cellular organisms. Such a system, in which homeoproteins act as morphogens, would have progressively gained in robustness through the recruitment of additional modes of signal transduction based on the interaction of growth factors and receptors, possibly under homeoprotein transcriptional and translational control. In support of this idea, it was recently demonstrated that homeoprotein Engrailed-2 can guide the axons of retinal ganglion cells following its internalization into the cones, where it regulates the translation of local mRNAs [50]. Engrailed may thus have been used as a signaling molecule prior to the invention of classic signaling mechanisms, leading to its present capacity to act as both a guidance molecule through translation regulation and a transcription factor for the expression of classic guiding cues [51-54]. We speculate that the same concept could apply to other messenger homeoproteins, including Pax6, and that this unforeseen Pax6 property may participate in both the definition of the eye field and the exchange of information between the retina and the lens.

## Conclusion

Expressing or addressing *in vivo *monoclonal antibodies against Pax6 modifies the pattern of *Pax6 *expression in the developing eye field and induces strong eye phenotypes affecting the retina and the lens. Based on the fact that Pax6 intercellular transfer is antagonized by the antibodies, we propose that Pax6 has two activities. A first cell autonomous activity, classically described for all transcription factors, is responsible for the control of the differentiation programs of the retina and lens, including the synthesis of *bona fide *growth factors and receptors. A second activity requiring Pax6 secretion is necessary for the full development of the eye anlage and supports the idea that several homeoproteins are morphogens with direct non-cell autonomous activity.

## Materials and methods

### Plasmids and proteins

Single chain antibody recombinants were prepared from total RNA of anti-Pax6 and anti-Engrailed 4G11 Hybridoma obtained from the Developmental Studies Hybridoma Bank (DSHB, University of Iowa and NICHD, Department of Biological Sciences, Iowa City, IA, USA). Cloning was achieved as in [55]. The coding sequences were inserted in a derivative of pSecTagHygroB (Invitrogen, Cergy, France) to give psecaP6 and psec4G11, while the signal peptide coding sequence was removed from pCBaP6 and pCB4G11; the open reading frames were also transferred downstream of the EF1a and T7 promoter (in a derivative of pEF1mycHis; Invitrogen) to give pEFsaP6 and pEFs4G11 (with signal peptide) and pEFaP6 and pEF4G11 (without signal peptide). The amino-terminal domain of Pax6 (coding sequence taken from pmPax6, a gift of T Czerny, Wien, Austria) was produced in bacteria and purified using poly-histidine extension. Full-length Pax6 protein, with a Hemaglutinin (HA) tag at its amino terminus, was expressed in COS-7 cells from the pCHAPax6 plasmid, under the control of the CMV promoter. Nucleic acids and protein manipulations were performed according to standard procedures. Construction details are available upon request.

### Intercellular transfer of Pax6

COS-7 cells, cultured in DMEM/F12 plus 10% fetal calf serum, were transfected with pCHAPax6 and either pEFs4G11 or pEFsaP6, using lipofectamin 2000 (Invitrogen). Six hours after transfection, cells were collected by trypsin treatment and plated on coated glass coverslips (15 μg/ml poly-ornithin) at a concentration of 2.5 × 10^3 ^cells/cm^2 ^together with HeLa cells (clone H2b, stably expressing a GFP-histone 2b fusion protein; a gift from Dr Valérie Doye, Institut Curie, Paris, France) at a concentration of 17 × 10^3 ^cells/cm^2^. In case of COS-7 cells singly transfected by pCHAPax6, anti-myc (9E10) or anti-Pax6 (both from DSHB) monoclonal antibodies were added (4 μg/ml) in the co-culture medium. After 24 hours, co-cultures were processed for immunocytochemistry as follows. Cells were washed and fixed (ethanol:acetic acid 93:7, 5 minutes, -20°C) and Pax6 expression was detected with an anti-Pax6 polyclonal antibody [56] followed by a Cy3-anti-rabbit antibody (Jackson Laboratories, Newmarket, UK). GFP was detected with an anti-GFP monoclonal antibody (Santa Cruz, Heidelberg, Germany) followed by an Alexa 488 anti-mouse antibody (Molecular Probes, Cergy, France).

Transfer was quantified for each condition using seven confocal sections processed with NIH Image J for signal analysis. Pax6 staining was quantified only in GFP positive cells (HeLa cells) in order to exclude signal from transfected COS cells.

### Fish embryo manipulation

Zebrafish (*Danio rerio*) were raised and staged according to [57]. RNA for single-chain antibodies transcribed *in vitro *from their linearized templates with the mMessage mMachine T7 kit (Ambion, Austin, USA)) were injected at a concentration of 100 ng/ml. Quantitative analysis of mRNA efficiency was performed on 50 microinjected embryos, manually dechorionated and freed from vitellus at the beginning of gastrulation, lysed in Laemmli buffer and electophoresed on a 12% SDS-polyacrylamide gel. Myc-tagged single-chain antibodies were detected on western blots with the 9E10 monoclonal anti-myc antibody (DSHB) and electrochimioluminescence (ECL, Amersham, Amersham Place, UK), and signal quantification was performed using Image J software. Hybridoma supernatants (anti-Pax6 and 9E10 anti-myc, DSHB) and FITC-anti-mouse IgG (Jackson ImmunoResearch, New Market, UK) were injected at mid-blastula stage according to Minchiotti and colleagues [58] at a concentration of 40 μg/ml.

### *In situ *hybridization, immunohistochemistry and morphometric analysis in zebrafish

Whole mount *in situ *hybridization and immunohistochemistry were performed according to standard protocols [59]. RNA probes were prepared from *Pax6a *(*Not*I, T7) and pCS2RX3 (*Hin*dIII, T7; a gift from Dr Laure Bally-Cuif, GSF, Neuherberg, Germany). Anti-myc antibodies were from DSHB (monoclonal 9E10) or Upstate Biotechnology (Charlottesville, USA) (polyclonal) and all secondary antibodies from Amersham. Embryos were embedded in 7.5% gelatin, 15% sucrose, 20 mM phosphate buffer pH 7.4, and frozen sections (14 μm) were mounted in 75% glycerol. Morphometric analysis was performed using Lucia G image-processing and analysis software (Version 4.61, Nikon, Kingston upon Thames, UK). All eyes were measured (arbitrary units) and individual measures were distributed among 12 size classes (8.3% increment steps), class 1 corresponding to the smallest eyes (as in Figure 3e).

## Competing interests

The author(s) declare that they have no competing interests.

## Authors' contributions

BL performed the experiments on zebrafish embryos, and wrote a first draft of the manuscript. AJ did the experiments on Pax6 passage in culture. AP conceived the study and designed it with BL and MV. MV and AP wrote the final version of the manuscript, which was approved by all authors.
